# Short-Term Effects of Intradialytic Hypoxemia on Plasma Markers of Hypoxia

**DOI:** 10.3390/ijms27167207

**Published:** 2026-08-12

**Authors:** Joanna Korzycka, Katarzyna Pęczek-Bartyzel, Michał Nowicki

**Affiliations:** Department of Nephrology, Hypertension, Transplantation and Internal Medicine, Central University Hospital, Medical University of Lodz, Pomorska 251, 92-213 Lodz, Poland

**Keywords:** hemodialysis, intradialytic hypoxemia, hypoxia-inducible factor, sirtuins

## Abstract

Short episodes of intradialytic hypoxemia (IDH) are a common but still under-recognized complication of hemodialysis with clinically significant consequences. The aim of the study was to analyze the effect of IDH on the secretion of hypoxia-inducible factors 1 and 2 and sirtuin 1. The study group consisted of 49 chronic hemodialysis patients, mean age 58.8 ± 15.3 years. Pulse oximetry was continuously recorded throughout hemodialysis. Blood was collected three times during a single mid-week hemodialysis session and at the start of the next mid-week session to assess levels of sirtuin 1 (SIRT1), hypoxia-inducible factor 1 (HIF-1), and hypoxia-inducible factor 2 (HIF-2). IDH, defined as a decrease in blood oxygen saturation below 90%, occurred in 22 (45%) patients. The mean time of hypoxemia during dialysis was 0.52% of the total hemodialysis session time. Plasma SIRT1 and HIF-2 concentrations did not change significantly during hemodialysis, whereas plasma HIF-1 levels decreased significantly. Multiple regression analysis showed that pre-dialysis plasma SIRT1 and HIF-1 explained a significant portion of the variability of blood oxygen saturation during hemodialysis. Pre-dialysis plasma levels of sirtuin 1 and HIF-1 could be considered predictors of the decline in blood oxygen saturation during hemodialysis sessions; they also determine the degree of variability of this parameter during the procedure.

## 1. Introduction

Transient intradialytic hypoxemia (IDH) is a common phenomenon occurring during hemodialysis treatment [1]. Despite short-term episodes of IDH only about one tenth of these patients develop prolonged intradialytic hypoxemia (PIH) characterized by an episode of hypoxemia lasting more than one-third of the hemodialysis session [1,2]. Transient IDH has been associated with clinically significant complications, including those significantly related with an inflammatory phenotype, increased risk of hospitalization, and mortality [1,2]. Patients with PIH have also been shown to have a greater need for erythropoiesis-stimulating factors and lower blood hemoglobin levels, which may indicate their hyposensitivity to renal anemia treatment [1,2]. In addition, hypoxia could increase the risk of serious cardiovascular events, deteriorate wound healing, and activate pro-inflammatory pathways [1,2].

Sirtuins are ubiquitous proteins, nicotine adenine dinucleotide(+)-dependent histone deacetylases [3]. They regulate various key signaling pathways. Increasing evidence from experimental studies confirms their role in regulating several complex physiological processes occurring in the kidneys, including the response to oxidative stress and production of erythropoietin [3]. In addition, sirtuins have cytoprotective effects and may be involved in the regulation of both blood pressure and sodium balance [2,4]. Sirtuin-1 (SIRT1) expression markedly increases during starvation and nutrient deprivation or when cells are exposed to oxidative stress and DNA damage [4,5].

The hypoxia-inducible factor (HIF) family of molecules are protein heterodimers that consist of α subunit (HIF-1α, HIF-2α, or HIF-3α) and β subunit [6,7]. HIF-2 is an oxygen-sensitive heterodimeric transcription factor that plays a key role in the cellular response to hypoxemia [6,7,8]. Both HIF-1 and HIF-2 stimulate oxygen delivery and promote cellular adaptation to hypoxia by stimulating erythropoiesis, angiogenesis, anaerobic glucose metabolism, iron metabolism, adenosine, and nitrogen oxide (NO) metabolism [6,7]. SIRT1 and HIF exhibit a number of physiological interactions, suggesting their common role in cellular metabolism. SIRT1 modulates cells’ response to hypoxia by interacting with the α subunit of HIF-2. SIRT1 stimulates HIF-2 transcriptional activity by selectively deacetylating HIF-2 under hypoxic conditions. In contrast, SIRT1 was not found to have any effect on HIF-1 expression [5,9,10]. Both HIF-1α and HIF-2α are activated by HIF-1α, which is involved in the initial adaptation process to hypoxia, as its concentration increases rapidly and then decreases to the baseline level within 72 h [11]. The increase in HIF-2α concentration is slower [9,10]. It was also found that erythropoietin is produced in fibroblast-like cells in the kidney interstitium in a HIF-2α-dependent manner [8]. The ability of SIRT1 to modify the transcriptional activity of HIF-2 under conditions of reduced oxygen pressure is particularly evident in patients with reduced SIRT-1 expression, in which case an increase in erythropoietin levels in response on hypoxemia may be impaired [5,10].

HIF-1 and SIRT1 form a regulatory axis that determines the response to changes in blood oxygenation. HIF-1 functions as a transcriptional regulator of the cellular response to oxygen deprivation, whereas SIRT1 acts as an NAD+-dependent protein deacetylase. SIRT1 binds directly to the HIF-1α subunit, deacetylating and inactivating it. Under conditions of oxygen, reduced intracellular NAD+ levels or metabolic changes lead to decreased SIRT1 activity, which inhibits deacetylation and releases HIF-1α [5,9,10].

The aim of the study was to investigate the effect of hemodialysis-related hypoxemia on the secretion of molecules that may counteract the effects of hypoxemia such as HIFs and SIRT-1. We postulated that short episodes of intradialytic hypoxemia occurring during hemodialysis may lead to increased HIF secretion and transient inhibition of SIRT1 activity.

## 2. Results

Forty-nine patients completed the study (24 women and 25 men). The mean age of the patients was 59 ± 15 years. The clinical characteristics of the study population are presented in Table 1. Intradialytic hypoxemia occurred in 22 patients (45% of the study population). The mean time of intradialytic hypoxemia constituted 0.52% of total hemodialysis session time.

**Figure 1:**
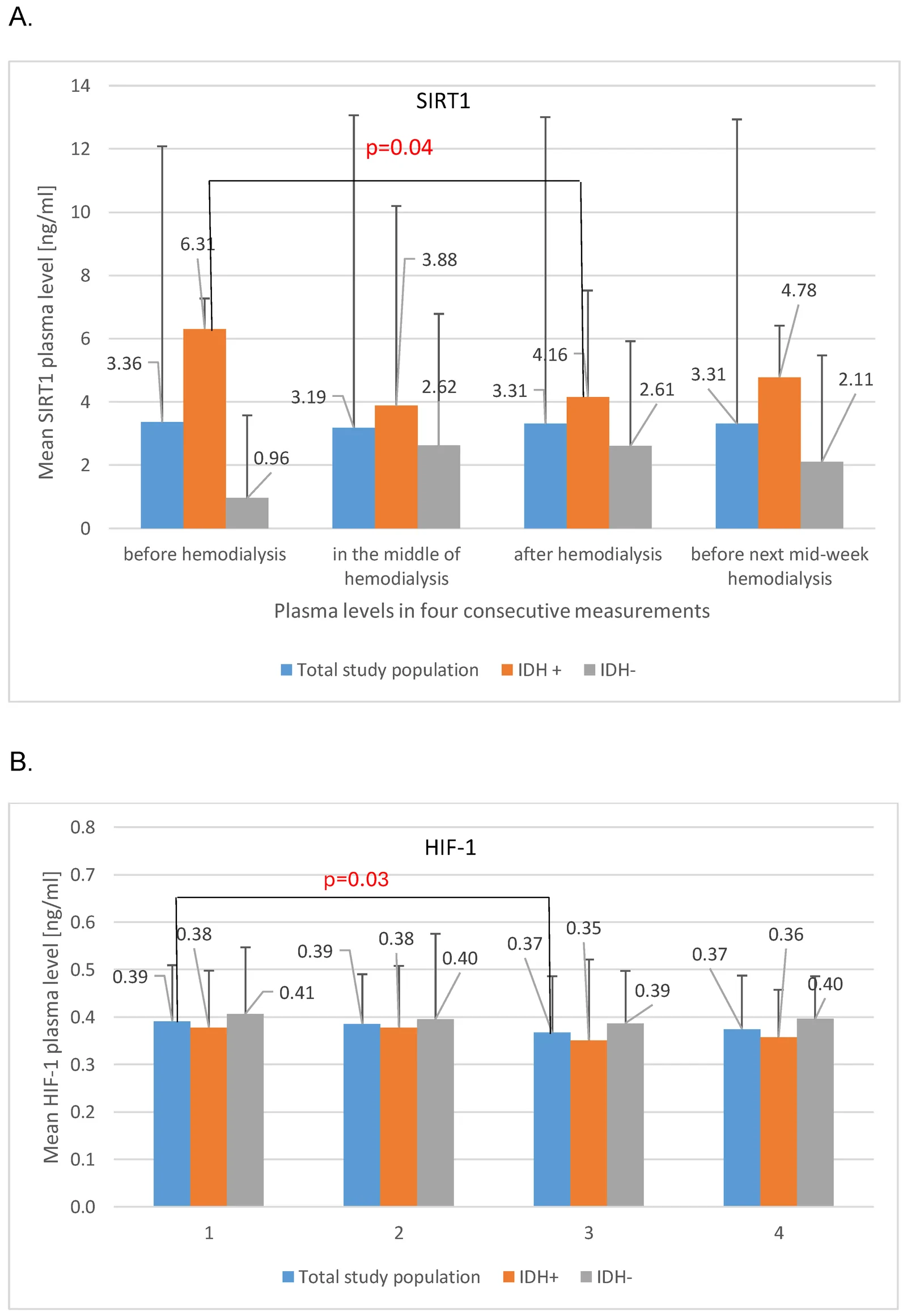

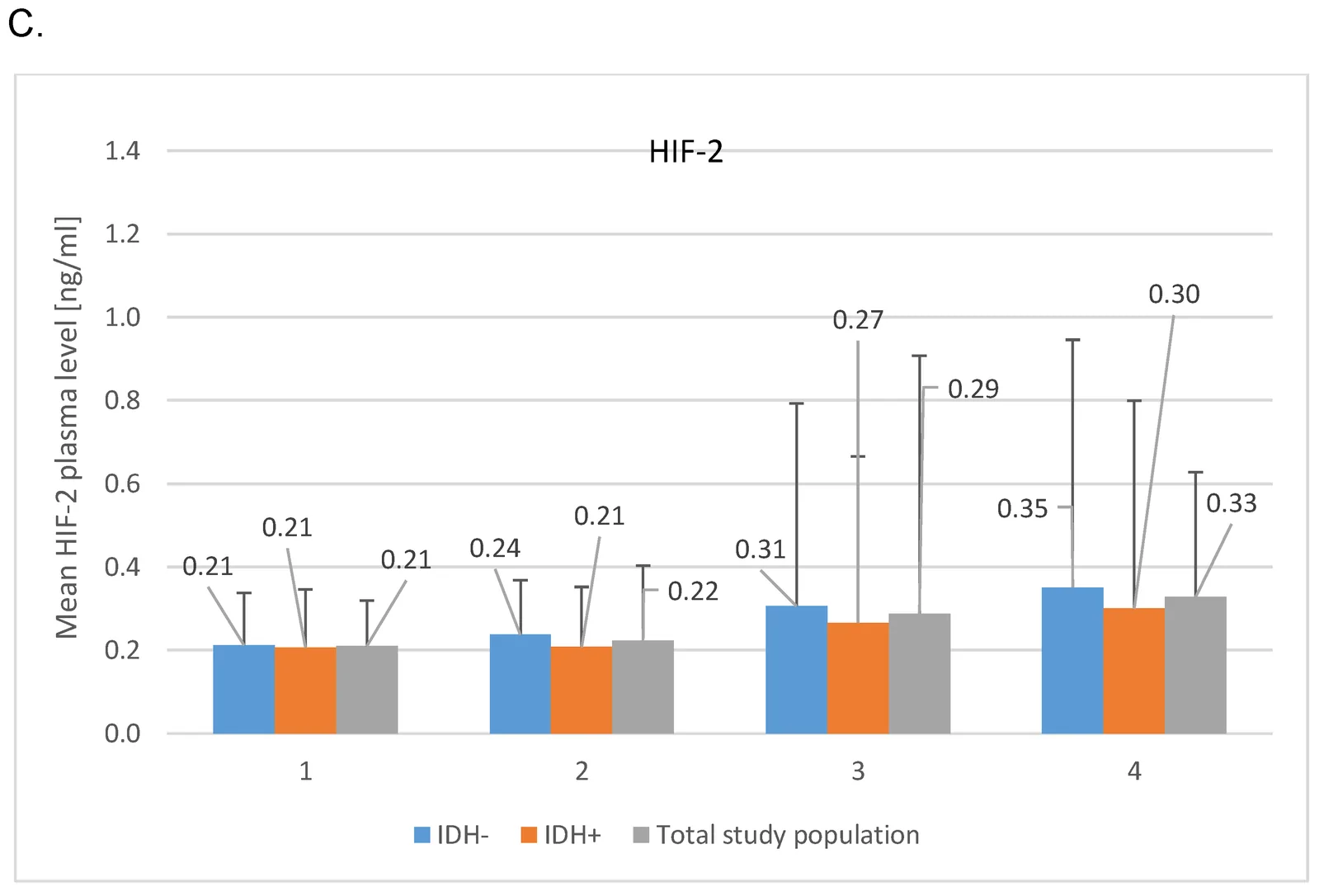
Mean plasma concentrations of SIRT1 (**A**), HIF-1 (**B**), and HIF-2 (**C**) measured before hemodialysis, at the midpoint of hemodialysis, after hemodialysis, and before the subsequent mid-week hemodialysis session, in the entire study group and in patients with (IDH+) and without (IDH−) intradialytic hypoxemia. All data were presented as the mean ± SD. IDH+—patients with intradialytic hypoxemia; IDH−—patients without intradialytic hypoxemia; SIRT1—sirtuin 1; HIF-1—hypoxia-inducible factor 1; HIF-2—hypoxia-inducible factor 2.

The study population was divided into two subgroups based on the occurrence of an IDH episode. Changes in plasma SIRT1, HIF-1, and HIF-2 levels in IDH-positive and IDH-negative subgroups are shown in Figure 1. Plasma SIRT1 and HIF-2 concentration did not significantly change during the hemodialysis session. In contrast plasma HIF-1 level decreased significantly during the hemodialysis session in mean measurements (Figure 1).

**Figure 2:**
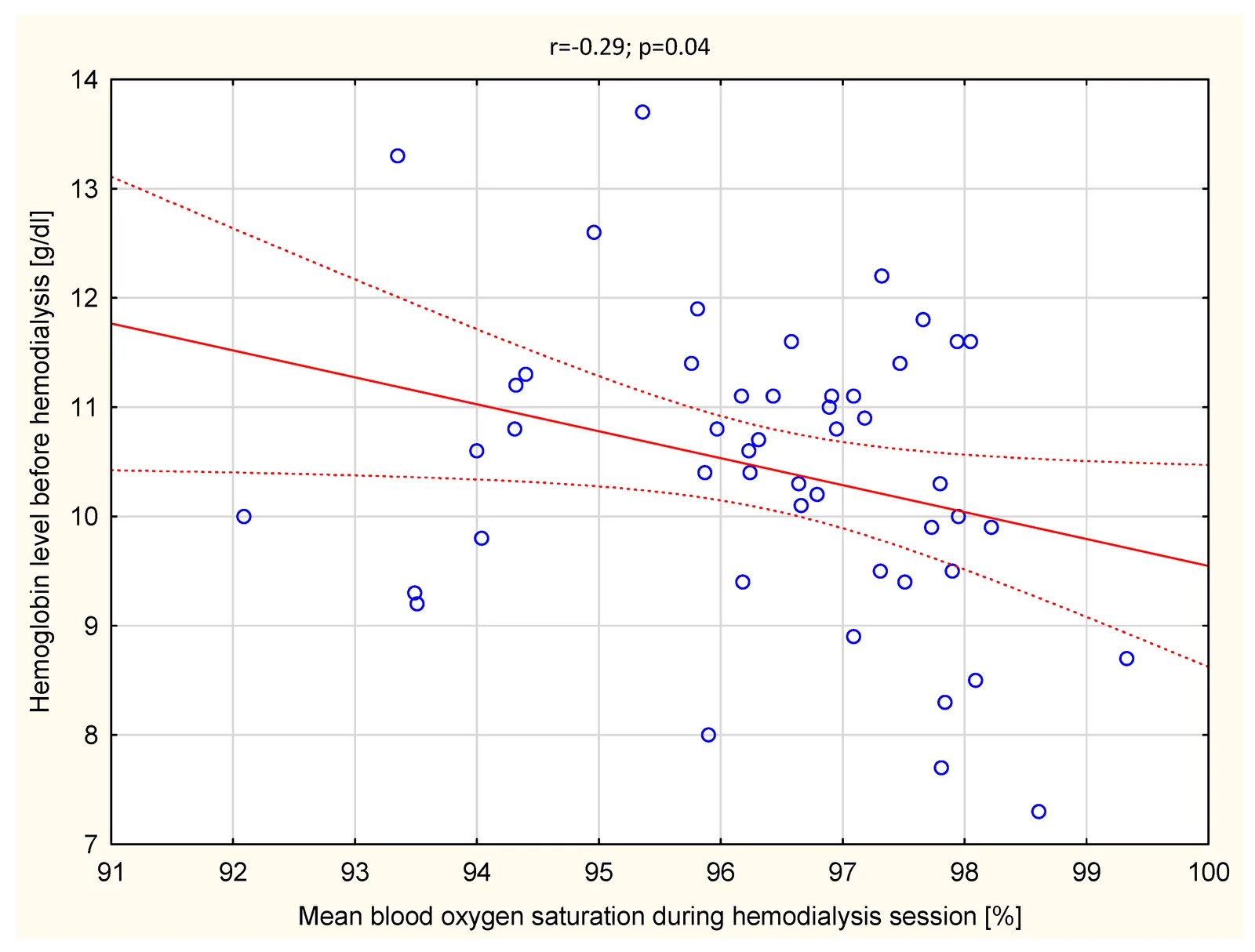
Linear correlation between mean baseline hemoglobin concentration and mean blood oxygen saturation during hemodialysis.

**Figure 3:**
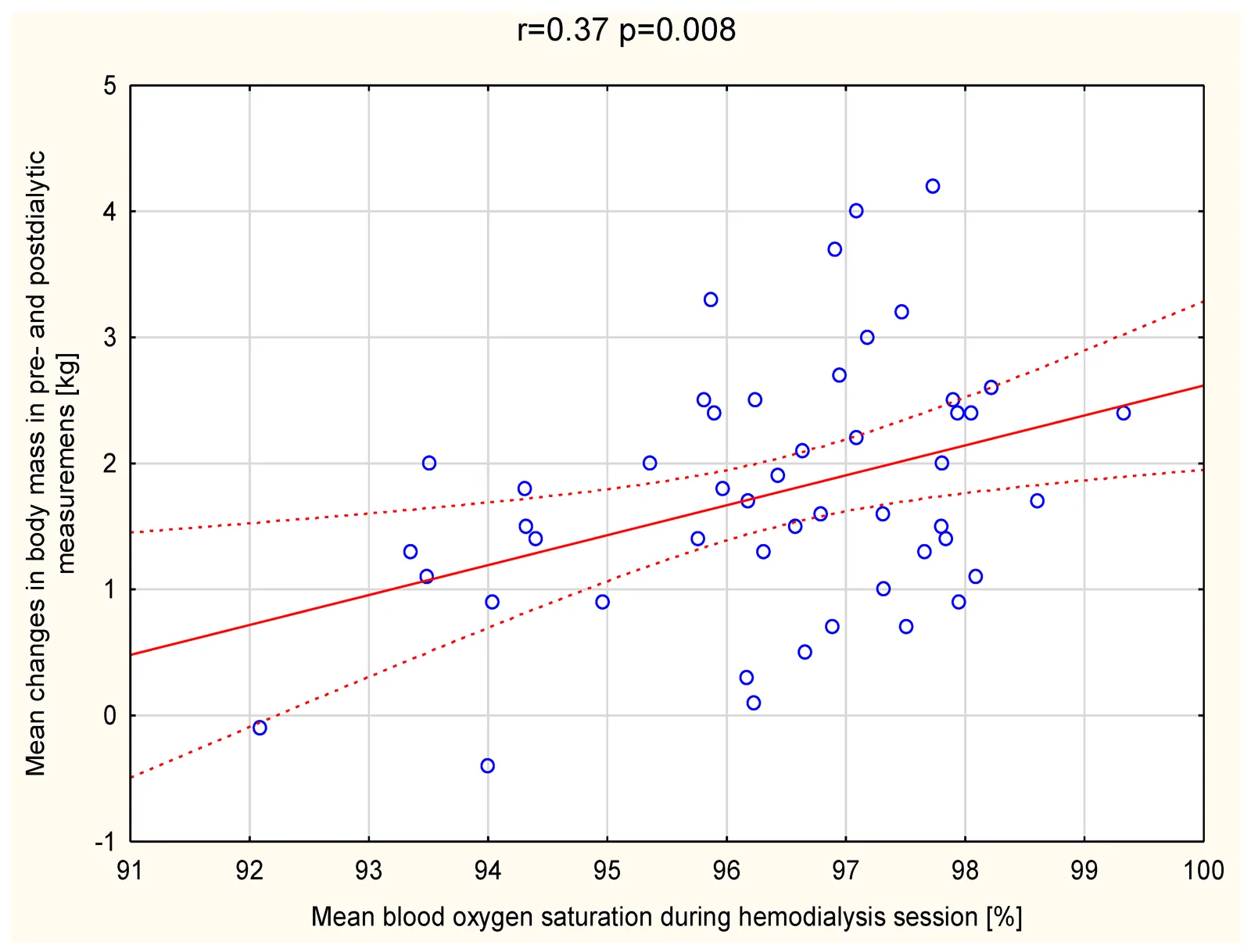
Linear correlation between mean intradialytic changes of body mass and mean blood oxygen saturation during hemodialysis session.

**Figure 4:**
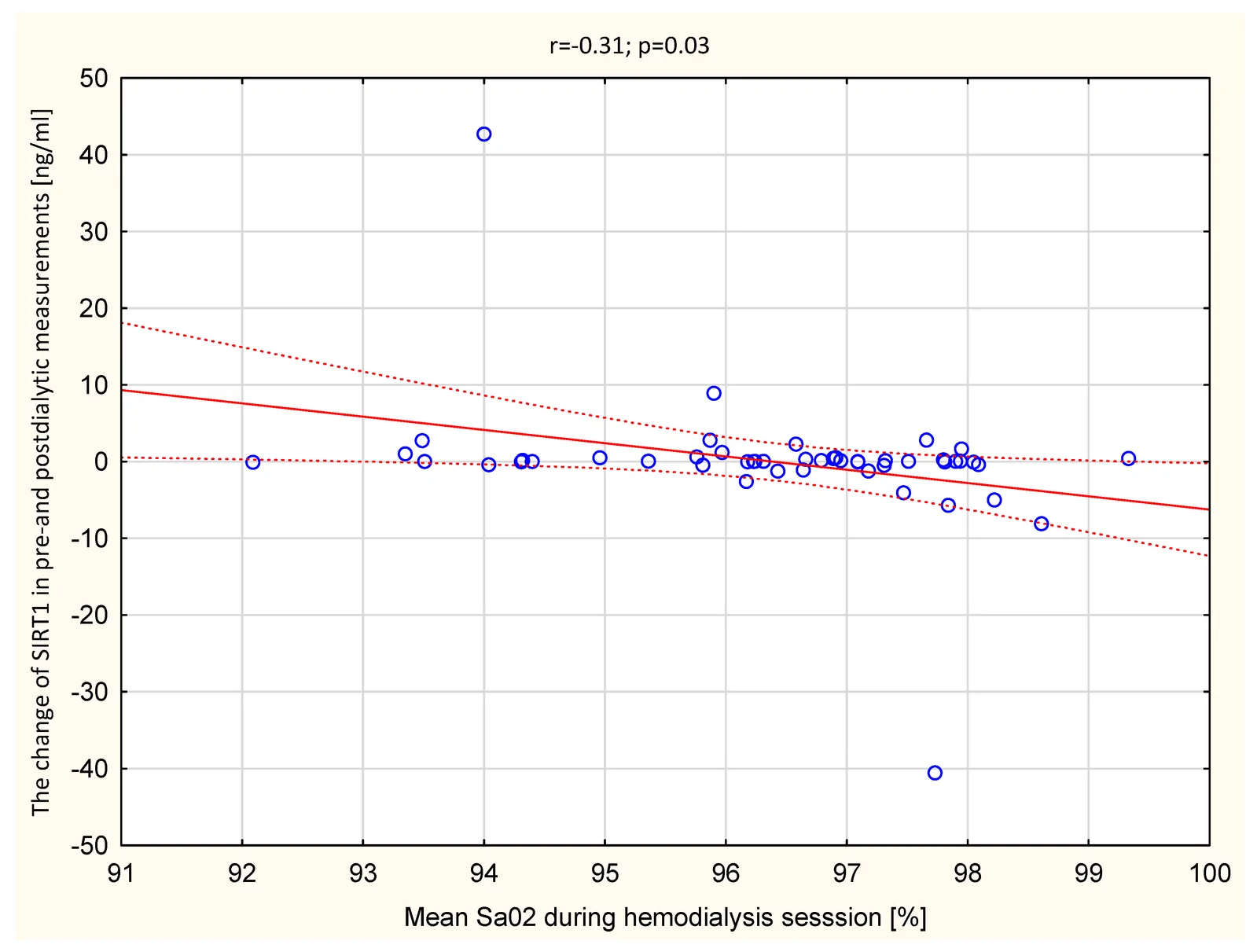
Linear correlation between mean blood oxygen saturation during hemodialysis and the change of sirtuin 1 plasma levels during hemodialysis session. SIRT1—sirtuin 1; SaO2—oxygen saturation.

A significant negative correlation was found between pre-dialysis hemoglobin concentration and mean blood oxygen saturation during the hemodialysis session (r = −0.29; *p* = 0.04) (Figure 2). No significant relation was found between the volume of ultrafiltration during hemodialysis and mean SaO2. However, the changes of body mass in pre- and post-dialytic assessments (ultrafiltration volume) and mean SaO2 were positively related (r = 0.37; *p* = 0.008) (Figure 3). Also the negative correlation between the mean SaO2 and the change of SIRT1 during hemodialysis session was revealed (r = −0.31; *p* = 0.03) (Figure 4). A statistically significant positive correlation was observed between the coefficient of variation of oxygen saturation and the concentration of SIRT1 in plasma before, during, and after hemodialysis (r = 0.28 *p* = 049, r = 0.31 *p* = 0.03, r = 0.25 *p* = 0.013, respectively).

There was also a significant negative correlation between the mean volume of ultrafiltration during hemodialysis and the mean intradialytic change of plasma HIF-1 level (r = −0.29; *p* = 0.046).

No statistically significant linear correlations were found between the changes in serum SIRT1, HIF-1, and HIF-2 and the volume of ultrafiltration or baseline Hb concentration in the studied groups.

Table 2 shows the results of the multiple regression analysis in which the coefficient of variation of oxygen saturation was selected as the dependent variable. Based on multiple regression analysis, it was found that baseline plasma concentrations of SIRT1 and HIF-1 may determine the variability of blood oxygen saturation during hemodialysis.

## 3. Discussion

Our study confirmed the relatively frequent occurrence of short episodes of IDH during hemodialysis sessions.

Despite the significant clinical importance of this phenomenon, only a few studies have been conducted so far on intradialytic hypoxia. Ban et al. suggested a relationship between episodes of low oxygenation and sleep apnea [12]. Other studies, like the one of Meyring-Wösten et al. indicated that long-term intradialytic hypoxemia was associated with a higher incidence of all-cause hospitalization and mortality during a 12-month follow-up [1]. In our study, we could not investigate these effects due to the different aim and design, which was limited to the analysis of changes in hypoxia markers during and after the hemodialysis session. However, we were able to demonstrate a significant decrease in plasma HIF-1 levels during hemodialysis, which may suggest excessive secretion of these molecules under hypoxic conditions, probably to protect cells from potential negative consequences [13]. That may correspond with the results of some recent studies investigating the role of the HIFs that may not only be responsible for minimizing the negative effects of hypoxia, but also may protect the cells against low oxygen availability [9]. Hao et al. and Liu et al. described the stimulatory effect of SIRT1 on the activity of HIF-2 and, consequently, the induction of a response to hypoxia and the independent effect of HIF-1 released in the body during hypoxemia [5,11]. Despite the description of the stimulatory effect of SIRT 1 on HIF 2 transcriptional activity through selective deacetylation under hypoxic conditions, no significant correlation between these molecules was observed in our study [13]. This phenomenon may be explained by a short duration of the episodes of hypoxemia that might have not been able to induce any significant changes of plasma sirtuin-1 concentration. Moreover, the differing kinetics of HIF-1 and HIF-2 molecules may influence the dynamics of changes in plasma levels. HIF-1α concentration increases significantly and rapidly—within 1–4 h—following a hypoxic episode. In contrast, HIF-2α levels rise slowly, forming a plasma peak over the course of ten to several dozen hours [11].

Abebe et al. suggested that the calculated SpO_2_ coefficient of variation allows for the analysis of the variability of this parameter [14]. In our study we assessed the associations of CoV of oxygen saturation and the baseline values of SIRT1, HIF-1, and HIF-2. Uzun et al. suggested that the assessment of the parameters related to body oxygenation may be of key importance for the early detection of complications of intradialytic hypoxemia in patients in critical condition [15]. These authors used central venous oxygen saturation (ScvO_2_), partial pressure of oxygen (PaO_2_), and perfusion index (PI) defined as the ratio of the pulse wave of the pulsatile portion (arteries) to the non-pulsatile portion (venous and other tissues) and showed that only in the patients who later died, a significant decrease in both PaO_2_ and ScvO_2_ was observed [15].

The strength of our study is the continuous recording of oxygen saturation during hemodialysis sessions. Plasma SIRT-1, HIF-1, and HIF-2 were also repeated four times, including an additional assessment at the start of the next mid-week hemodialysis session. In our study, a finger pulse oximeter was used to measure blood oxygen saturation as it is a non-invasive, inexpensive, and easy to perform method. The weakness of this method is the possible interference of the measurement result by factors such as low peripheral blood flow and large decreases of systolic blood pressure below 80 mm Hg, severe limb edema, or dyshemoglobinemia [16].

## 4. Materials and Methods

The study was prospective and observational. The study protocol was approved by the local Bioethics Committee (No. RNN/112/21/KE). All study participants gave written informed consent to participate. The study was conducted strictly in accordance with the principles of biomedical research ethics contained in the Declaration of Helsinki. Forty-nine chronic hemodialysis patients participated in the study. The study included patients who were over 18 years of age, undergoing hemodialysis 3 times a week and remaining in a stable general health condition for the last 3 months. All patients included in the study were treated with darbepoetin alfa, which was dosed once every two weeks strictly according to the KDIGO and ERBP recommendations. The study was performed during the week when darbepoetin alfa was not administered. Exclusion criteria were as follows: elevated inflammatory serum parameters or any clinical signs of inflammation, respiratory failure with predialysis blood oxygen saturation < 94%, active cancer, psychiatric disorders or dementia, previous or ongoing use of hypoxia-inducible factor stabilizers, unstable hemoglobin concentration, and modification of erythropoiesis-stimulating factor or intravenous iron dose within the last 3 months.

All measurements were carried out during a mid-week hemodialysis session, i.e., depending on the dialysis shift either on Wednesday or Thursday. Blood oxygen saturation was continuously recorded during the whole hemodialysis session with the pulse oximeter (OEM UN-S1 Handheld Pulse Oximeter device, Wuhan UN—Medical Technology, Wuhan, China). IDH was defined as short-term hypoxemia with reduced blood oxygen saturation (SpO_2_) below 90% [1,2]. Additionally, blood samples were collected 4 times, that is, at the beginning, in the middle, and at the end of hemodialysis and at the beginning of the next mid-week hemodialysis session to assess plasma concentration of SIRT1, HIF-1, and HIF-2. The blood samples were centrifuged at 3–4 °C 2000× *g* for 10 min, then aliquots were stored in a deep freezer at −70 °C for no longer than 6 months. After all samples were collected, laboratory measurements were performed in a single batch by a qualified technician. ELISA tests were used for analyses: Peachtree Corners, GA 30092, USA; RayBio^®^ Human Sirtuin 1 ELISA Kit for Cell Culture Supernatants, Plasma, and Serum samples. La Jolla, CA 92038, USA; AAA Biotech, Human HIF-1alpha (Hypoxia Inducible Factor 1 Alpha) ELISA Kit; Human Hypoxia-inducible factor 2 (HIF-2) ELISA Kit.

Statistical analysis was performed using Statistica version 13.0 PL (TIBCO Software Inc., Palo Alto, CA, USA). The results for normally distributed variables are presented as mean ± standard deviation (SD) and for normally distributed variables as median with interquartile range. To analyze associations between pairs of variables, Pearson’s or Spearman’s correlation coefficients were calculated. A *p*-value of <0.05 was considered statistically significant. A multiple regression model with the forward stepwise selection was used. The modeling process began with an empty model, adding statistically significant variables (*p* < 0.05) in subsequent stages. The coefficient of variation of oxygen saturation values was taken as the dependent variable. The predicting variables included in the model were baseline blood hemoglobin and plasma levels of SIRT1, HIF-1, and HIF-2.

## 5. Conclusions

Our study did not demonstrate any significant correlation between the parameters of the red blood cell system and the variability of hypoxemia markers and the incidence of intradialytic hypoxemia. The assessment of pre-dialysis sirtuin-1 level may serve as a predictor of clinically significant decreases of blood oxygen saturation during a hemodialysis session. SIRT1 plasma level is associated with oxygen saturation changes during hemodialysis. SIRT1 and HIF-1 baseline plasma levels could determine the variability of oxygen saturation during a hemodialysis session.

## Figures and Tables

**Table 1:** Baseline clinical characteristics of the study group.

	Mean	SD	Median	Minimum	Maximum
Age [years]	58.8	15.3	64	26	79
Calcium plasma level [mmol/L]	2.3	0.2	2.3	1.9	3.1
Coefficient of variation of saturation [%]	1.4	0.8	1.2	0.5	4.2
CRP plasma level [mg/L]	6.30	4.9	5.5	0.4	24.9
Hemoglobin concentration [g/dL]	10.4	1.34	10.6	7.3	13.7
Mean blood oxygen saturation before hemodialysis [%]	96.9	1.49	97	94	99
Mean intradialytic body mass gain [kg]	1.95	0.85	2.1	0	3.6
Mean urea plasma level before hemodialysis [mmol/L]	23.0	4.9	22.2	11.4	35.0
Mean urea plasma level after hemodialysis [mmol/L]	9.1	3.4	8.6	3.8	24.4

CRP—C-reactive protein. SD—standard deviation.

**Table 2:** Multiple regression model with the mean coefficient of variation of oxygen saturation as a dependent variable.

R = 0.48 R^2^ = 0.23 *p* < 0.04 Estimation Error: 0.71				
Independent Variables	Regression Coefficient	Standard Error	t	*p*-Value
Baseline sirtuin 1 mean plasma level [ng/mL]	0.37	0.14	2.65	0.01
Baseline hypoxia-inducible factor 1 mean plasma level [ng/mL]	0.32	0.14	2.27	0.03
Baseline hypoxia-inducible factor 2 mean plasma level [ng/mL]	−0.09	0.14	0.57	0.5
Baseline hemoglobin concentration [g/dL]	0.27	0.08	1.91	0.8

## Data Availability

All data generated or analyzed during this study are included in the paper. Further inquiries can be directed to the corresponding authors.
